# Auxin and cytokinin regulate growth dynamics underlying carpel initiation in Arabidopsis

**DOI:** 10.1093/jxb/eraf535

**Published:** 2025-12-12

**Authors:** Andrea Gómez-Felipe, Stefan de Folter, Daniel Kierzkowski

**Affiliations:** Département de Sciences Biologiques, Institut de Recherche en Biologie Végétale, Université de Montréal, 4101 Sherbrooke St E, Montréal, QC H1X 2B2, Canada; Centro de Investigación y de Estudios Avanzados del Instituto Politécnico Nacional (Cinvestav), Unidad de Genómica Avanzada (UGA-Langebio), Irapuato CP 36824, México; Département de Sciences Biologiques, Institut de Recherche en Biologie Végétale, Université de Montréal, 4101 Sherbrooke St E, Montréal, QC H1X 2B2, Canada; University of Sydney, Australia

**Keywords:** *Arabidopsis thaliana*, auxin, carpel development, cytokinin, primordium initiation, time-lapse imaging

## Abstract

Plant organ initiation requires precise spatial and temporal coordination of cellular behaviors. In Arabidopsis, the gynoecium, the female reproductive structure formed by two fused carpels, is initiated after the termination of the floral meristem. Proper initiation of the carpels is crucial for ovule protection, successful fertilization, and the formation of diverse fruit structures. While the phytohormones auxin and cytokinin are known to regulate organogenesis, their interplay during the earliest stages of carpel development is not fully understood. In particular, how auxin–cytokinin crosstalk influences growth patterns that shape the emerging gynoecium remains unknown. Here, we combined confocal live imaging, hormone treatments, and hormone reporters to capture at cellular resolution the dynamic processes of cell expansion and division that drive the onset of the carpel developmental program. We show that carpel primordia initiation is driven by growth differences between the fast-expanding peripheral and slow-growing central regions, transforming the dome-shaped floral meristem into two fused carpels connected by a continuous ring of cells. Ectopic cytokinin treatments increase cell growth and division to promote an increase in the size of the carpel primordia, whereas inhibition of auxin transport has the opposite effect. Our results suggest that the interplay between auxin and cytokinin is indispensable for establishing the correct organ geometry during carpel initiation.

## Teaser

Live imaging and manipulation of hormones shows how auxin–cytokinin crosstalk drives differential growth at the cellular level in the Arabidopsis gynoecium to establish the correct geometry for carpel primordia initiation.

## Introduction

Organ primordia are small clusters of undifferentiated cells that generate lateral plant organs such as leaves and flowers. Their formation, growth, and patterning are governed by interconnected gene-regulatory networks, hormone crosstalk, and environmental cues (Reinhardt *et al*., 2003; Heisler *et al*., 2005; Besnard *et al*., 2014a, 2014b; Landrein *et al*., 2018; Mohammed *et al*., 2018; Burian *et al*., 2022; Sluis and Hake, 2015). Dissecting how primordia coordinate cell divisions, cell expansion, and fate specification is therefore central to understanding organ initiation and morphogenesis.

In Arabidopsis, the innermost floral organs, the two carpels, originate from a dome-shaped outgrowth that emerges from the center of the floral meristem (FM), and meristematic activity of the FM ceases at late stage 5 (Smyth *et al*., 1990; Roeder and Yanofsky, 2006). By stage 7, the primordia elongate and fuse along their medial flanks to form the carpel margin meristem, a bilaterally symmetric structure that gives rise to ovules and internal tissues such as the septum and transmitting tract (Nole-Wilson *et al*., 2010; Reyes-Olalde *et al*., 2013; Reyes-Olalde and de Folter, 2019). This structure ultimately develops into the gynoecium, the female reproductive structure, from which the fruit is derived (Roeder and Yanofsky, 2006; Herrera-Ubaldo and de Folter, 2022).

Genetic and hormonal pathways converge to direct gynoecium development and patterning (Nemhauser *et al*., 2000; Ståldal, *et al*., 2008; Sorefan *et al*., 2009; Sundberg and Østergaard, 2009; Marsch-Martínez *et al*., 2012; Zúñiga-Mayo *et al*., 2019). Auxin and cytokinin cooperate but also antagonize one another to position and shape the carpel primordia (Zúñiga-Mayo *et al*., 2014; Marsch-Martínez and de Folter, 2016; Müller *et al*., 2017). During early stage 5, auxin accumulates in two distinct foci at the periphery of the FM within the epidermal (L1) layer, corresponding to the sites of carpel primordia initiation (Larsson *et al*., 2014; Müller *et al*., 2017). These hormone response patterns are achieved through the coordinated action of auxin synthesis and transport (Larsson *et al*., 2014; Moubayidin and Østergaard, 2014; Müller *et al*., 2017).

Auxin locally represses cytokinin signaling through ARABIDOPSIS HISTIDINE PHOSPHOTRANSFER PROTEIN 6 (AHP6) in the lateral domains (the future carpel primordia), whereas cytokinin promotes auxin biosynthesis via YUCCA1 and TRYPTOPHAN AMINOTRANSFERASE OF ARABIDOPSIS 1 (TAA1), and facilitates its transport through PINFORMED3 and 7 in the medial domain (Müller *et al*., 2017; Reyes-Olalde *et al*., 2017). However, the downstream cellular consequences of this hormonal crosstalk, particularly its impact on growth dynamics, remain unknown. Classical histology has revealed static features such as cell numbers and tissue architecture during carpel primordia formation (Hill and Lord, 1989; Crone and Lord, 1994; Jenik and Irish, 2000), but spatiotemporal patterns of differential growth, cell proliferation, and changes in tissue geometry have yet to be quantified.

Technical limitations account for much of this gap in our knowledge. Early carpel primordia are tiny and are quickly embedded inside the floral buds, preventing live imaging and making it difficult to examine with serial sectioning. In addition, hormone activity during early stages has been visualized using various reporters, but imaging limitations have prevented a clear picture of auxin and cytokinin distribution during carpel primordium initiation (Larsson *et al*., 2014; Müller *et al*., 2017). Although recent advances in live imaging and 3D segmentation now allow tracking of growth in real time at cellular resolution (Fernandez *et al*., 2010; Kierzkowski *et al*., 2012; Le Gloanec *et al*., 2022; Min *et al*., 2022; Singh Yadav and Roeder, 2024; Silveira *et al*., 2025), quantitative imaging of carpel initiation in Arabidopsis, and the link between key molecular regulators and cell behavior, are still lacking.

In this study, we combined confocal live imaging of carpel primordia establishment in Arabidopsis with chemical perturbations to examine how growth patterns and signaling interactions drive the earliest stages of carpel development. We focused on how auxin and cytokinin influence the spatial and temporal growth dynamics at cellular resolution, and how early asymmetries contribute to the establishment of medial and lateral domains, including a potential pre-pattern for the carpel margin meristem.

## Materials and methods

### Plant material and growth conditions

The transformants *DR5v2::nls-3xVenus* (Liao *et al*., 2015), *pTCSn::GFP* (Zürcher *et al*., 2013), *pUBQ10::myr-YFP* (Willis *et al*., 2016), and *pUBQ10::myr-TdTomato* (Melnyk *et al*., 2015) are all in the *Arabidopsis thaliana* Col-0 background. *pUBQ10::myr-TdTomato* and *DR5v2::nls-3xVenus* were crossed and analysed in F3. *pUBQ10::myr-TdTomato* and *pTCSn::GFP* were crossed and analysed in F3. Seeds were germinated and subsequently maintained in soil in a growth chamber under long-day conditions of 16/8 h light/dark, 150 μmol m^–2^ s^–1^, 60–70% relative humidity, and a constant temperature of 22 ±1 °C.

### Live imaging of carpel initiation

Transgenic plants expressing *pUBQ10::myr-YFP* were used for live imaging of carpel initiation. Main inflorescences from 3-week-old plants were excised ∼2 cm below the apex. Floral buds older than stage 7 were removed using fine tweezers to expose young floral primordia at the shoot apex. Stage 4 floral buds were selected, and the median sepals were carefully removed using a needle and/or fine tweezers under a stereomicroscope.

Dissected floral meristems were transferred to Petri dishes containing half-strength Murashige and Skoog (½MS) medium supplemented with vitamins (Murashige and Skoog, 1962), 1% (w/v) agar, 1.5% (w/v) sucrose, and 0.1% (v/v) Plant Protective Medium (Plant Cell Technology). Samples were immersed in water supplemented with 0.1% (v/v) protective medium and imaged from the top at 24 h intervals.

After each imaging session, samples were returned to the controlled growth chamber under the long-day conditions described above.

### Cytokinin treatment

The synthetic cytokinin N6-benzylaminopurine (BAP; Sigma-Aldrich) was used at a final concentration of 10 μM. A 100 mM BAP stock solution was prepared in 100 mM NaOH and sterilized by filtration through a 0.2 μm filter. For treatment, the stock solution was added to cooled, autoclaved ½MS medium supplemented with vitamins, 1.5% (w/v) agar, and 1.5% (w/v) sucrose to reach a final BAP concentration of 10 μM.

Transgenic plants expressing *TCSn::GFP* and *DR5v2::NLS-3xVenus* were used for cytokinin response assays. Main inflorescence shoots from 3-week-old plants were dissected, and floral buds older than stage 7 were removed. The dissected inflorescences were then cultivated *in vitro* on 60 mm Petri dishes containing the prepared BAP-supplemented medium. Samples were grown under the long-day conditions described above.

### NPA treatment

The inhibitor of PIN-mediated auxin transport, N-1-naphthylphthalamic acid (NPA), was used at a final concentration of 2 μM. A 10 mM NPA stock solution was prepared in DMSO. For treatment, the stock solution was added to cooled, autoclaved ½MS medium supplemented with vitamins, 1% (w/v) agar, and 1.5% (w/v) sucrose to reach a final NPA concentration of 2 μM.

Transgenic plants expressing *TCSn::GFP* and *DR5v2::NLS-3xVenus* were used for cytokinin response assays. Main inflorescence shoots from 3-week-old plants were dissected, and floral buds older than stage 7 were removed. The dissected inflorescences were then cultivated *in vitro* on 60 mm Petri dishes containing the prepared NPA-supplemented medium and the samples were grown under the long-day conditions described above.

### Confocal microscopy

All the confocal images were captured using a Zeiss LSM 800 confocal laser-scanning upright microscope with a 40× water-dipping objective (W Plan-Apochromat 40×/1.0 DIC VIS-IRM27). Excitation was achieved using a diode laser with 488 nm for YFP and GFP, and 561 nm for TdTomato. Images were collected at 500–550 nm for YFP and GFP, and 600–660 nm for TdTomato. Between imaging, the samples were transferred to the growth chamber with standard long-day conditions described above. Images were acquired from top to bottom of each floral bud using a step size of 0.5 µm, at 16 bits image depth, 512×512 pixel resolution, with bidirectional mode, and no averaging.

### Image analysis

The time-lapse series acquired were segmented using the MorphoGraphX software (Barbier de Reuille *et al*., 2015; Strauss *et al*., 2019). To segment cells from the floral meristem to carpel initiation, the surface geometry of the meristem and carpel primordia were first extracted using YFP or TdTomato fluorescence signal as described previously (Kierzkowski *et al*., 2012). Briefly, stacks were blurred using Gaussian blur (values: 0.3/0.3/0.3 in X/Y/X) followed by edge detect (threshold 8000). The resulting binary image was used to create a mesh using a marching-cube algorithm (5 µm initially, subdivided three times). A portion of the YFP or TdTomato membrane signal (2–4 µm from the surface) was then projected onto the resulting curved floral meristem surface and auto-segmented into cells using the watershed technique (Strauss *et al*., 2019). After the segmentation, parent relations between each time-point were determined manually (Kierzkowski *et al*., 2019). The segmented images were used to calculate expansion, area, proliferation, and curvature for each cell. Area expansion was calculated as the relative increase between the surface area of a mother cell and the area of its daughter cell(s) at the next time-point, and is expressed as percentage of area increase: [(Total area of daughter cells at T1/Area of mother cell at T0)−1] ×100; where T0 and T1 are consecutive time-points. Cell division was quantified by counting the number of daughter cells at the next time-point, T2, that originated from a single mother cell at T1. Gaussian curvature heatmaps were generated by selecting all cells belonging to the carpel primordia and applying the ‘Compute Tissue Curvature’ tool with a radius parameter of 10 (Silveira *et al*., 2022). Based on the resulting curvature values, two regions were defined: the central region, corresponding to nearly flat or slightly concave areas (curvature −0.04 to 0.01), and the peripheral region, characterized by positive curvature values (0.02 to 0.04) that mark the bulging zones of the primordia. These regions were subsequently traced back to the initial time-point using a custom Python script, which followed parent–daughter cell relationships from the final to the initial time-point, enabling visualization of the corresponding cell populations over time (Kierzkowski *et al*., 2019). Parts of the plant tissue surrounding the floral meristem were removed digitally.

### Statistical analysis

All statistical analyses were performed in R version 4.4.2. To compare quantitative parameters (e.g. cell expansion, division, and size) between the central and peripheral regions, we applied a non-parametric Mann–Whitney *U*-test because the data did not meet normality assumptions and sample sizes were relatively small. To compare treatments, data were grouped by experimental factors including treatment group [wild type (WT), BAP, NPA], time-point (T0=0 h, T1=24 h, T2=48 h, T3=72 h), and tissue region (central region, peripheral region). To test for significant differences between treatment groups within each region and time-point, we performed a one-way ANOVA followed by Tukey’s Honest Significant Difference (HSD) post-hoc test.

## Results

### Growth differences between the central and peripheral regions underly gynoecium initiation

**Fig. 1.**
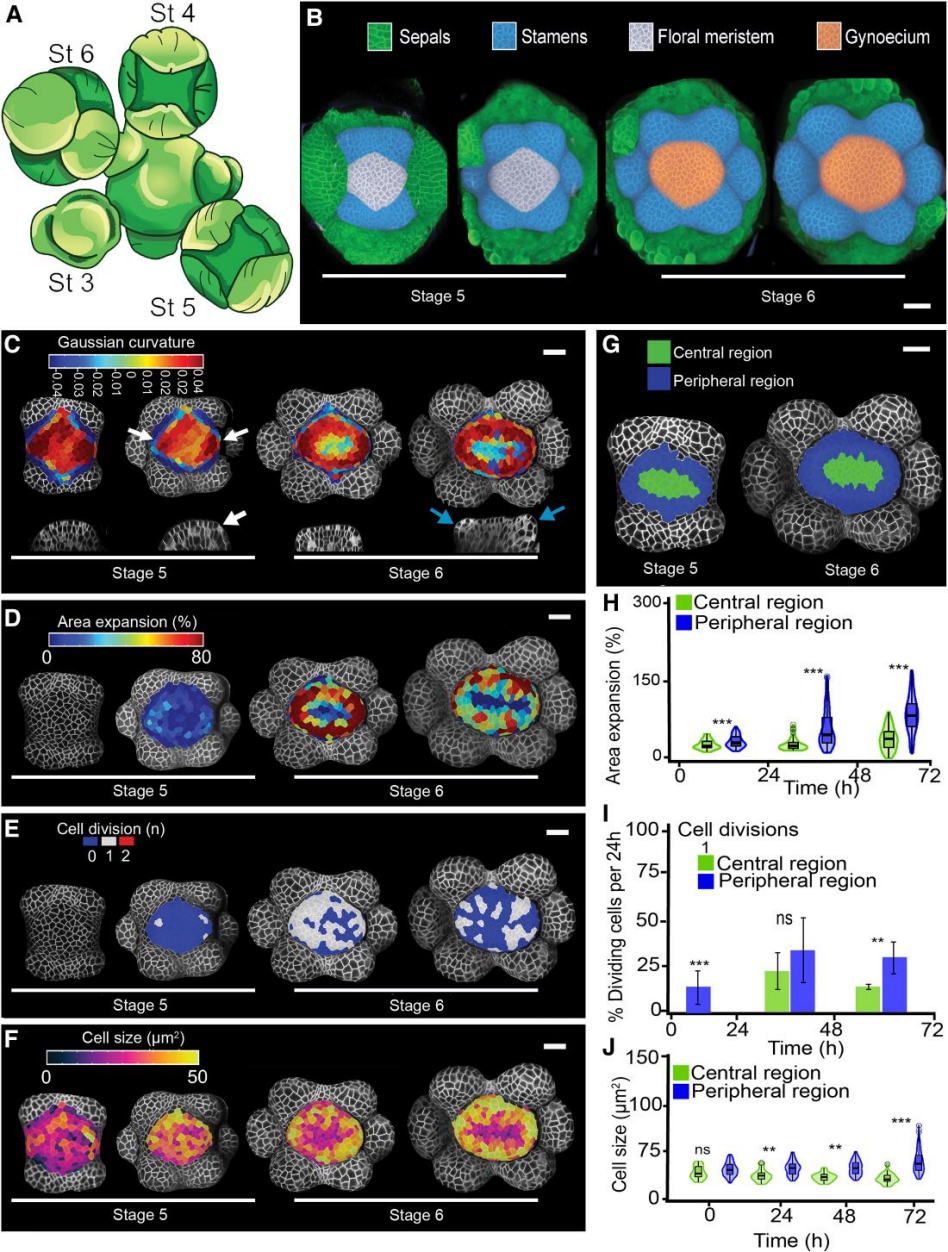
Growth differences between meristem regions underlies gynoecium initiation in Arabidopsis. (A) Schematic top view of the inflorescence showing initiating floral buds at different developmental stages (St) according to Smyth *et al*. (1990). (B) Time-lapse series capturing carpel primordia establishment from early stage 5 to late stage 6 over 72 h at 24 h intervals. Median sepals were removed. (C) Heat maps of Gaussian curvature at the different stages; the bottom row shows virtual longitudinal sections through the bud along its lateral axis. Increases in positive Gaussian curvature are indicated with white arrows (dark red zones in the top image). The blue arrows indicate a carpel primordium emerging as a lateral protrusion on the floral meristem. (D) Heat map of percentage area expansion between the stages. (E) Heat map of cell divisions between the stages. (F) Heat map of cell sizes at the different stages. (G) Lineage tracing of the central and peripheral regions from early stage 5 to late stage 6. Scale bars are 20 µm. (H–J) Quantification of (H) area expansion, (I) cell divisions, and (J) cell sizes; ‘1’ indicates that the cells underwent one division. The violin and box-plots represent the 95% confidence intervals and the means (*n*=3 time-lapse series; error bars are SD). The bar-plots show the percentage of cells that divided relative to the total number of cells in the central and peripheral regions per 24 h. Data from individual replicates are shown in Supplementary Fig. S1. Significant differences between regions were determined using Mann-Whitney *U*-tests: **P<0.001; ***P<0.0001; ns, not significant.

To uncover cellular growth patterns during carpel primordia initiation, we adapted a confocal live-imaging pipeline previously developed for stamen imaging in the model plant Arabidopsis (Silveira *et al*., 2022; Wang *et al*., 2025). We began imaging at early stage 5, when the floral meristem (FM) remains dome-shaped and no morphological or geometric signs of carpel initiation are yet visible, and continued until late stage 6, when two fused carpels connected by a continuous ring of cells were clearly established; stages 5 and 6 were further divided in early and late (Fig. 1A, B) (Smyth *et al*., 1990).

We found that ∼60±6 epidermal cells (*n*=3 meristems) at early stage 5 contributed to the gynoecium. Carpel primordia emerged as two lateral protrusions on the FM at late stage 6 (Fig. 1C, blue arrows), but the first signs of their initiation were already visible at late stage 5, with an increase in positive Gaussian curvature (Fig. 1C, white arrows). The increase in positive curvature became more pronounced during stage 6, coinciding with enhanced cell expansion (Fig. 1C, D). These differences in curvature and expansion allowed us to define two distinct regions within the developing primordia: a central region characterized by low growth and negative curvature, and a peripheral region exhibiting high growth and positive curvature (Fig. 1G). Growth then intensified across the entire periphery of the FM, whereas cells in the central region grew little at early stage 6 (Fig. 1D; Supplementary Figs S1A, B, G, H, S2A). This differential growth gradually reshaped the initially domed meristem into emerging carpel primordia characterized by a flattened central region and two rounded lateral ridges (Fig. 1C, bottom; Supplementary Video S1). By late stage 6, the slow-growing central region extended laterally and began to invaginate, creating areas of negative Gaussian curvature, while peripheral cells continued to expand (Fig. 1C, D, H). A few cell divisions were observed in the peripheral region at stage 5, while no divisions were detected in the central region (Fig. 1E, I; Supplementary Fig. S1C, D, I, J). By early stage 6, significantly higher rates of cell division were quantified in the peripheral region compared to the central region. At early stage 5, however, cell sizes were relatively homogeneous across the meristem, ranging from 15–42 µm^2^ in the central region and from 11–51 µm^2^ in the periphery (Fig. 1F, J; Supplementary Figs S1E, F, K, L, S2B). By stage 6, peripheral cells began to enlarge, reaching sizes between 14–94 µm^2^, while cells in the central region remained smaller (8–40 µm^2^), maintaining sizes comparable to those observed at stage 5. Taken together, these results indicated that the formation of fused carpel primordia is driven by differential growth and cell division between the peripheral and central regions of the early gynoecium.

### Cytokinin accelerates growth and division during gynoecium initiation

The activity of the plant hormone cytokinin is regulated by genes controlling its biosynthesis, degradation, and signaling pathways (Kieber and Schaller, 2018). Cytokinin influences various developmental processes, including the precise timing of organ initiation within the inflorescence meristem (Besnard *et al*., 2014a, 2014b). Thus, we next investigated how cytokinin affected growth dynamics during carpel primordia formation by treating FMs with the synthetic cytokinin BAP.

**Fig. 2.**
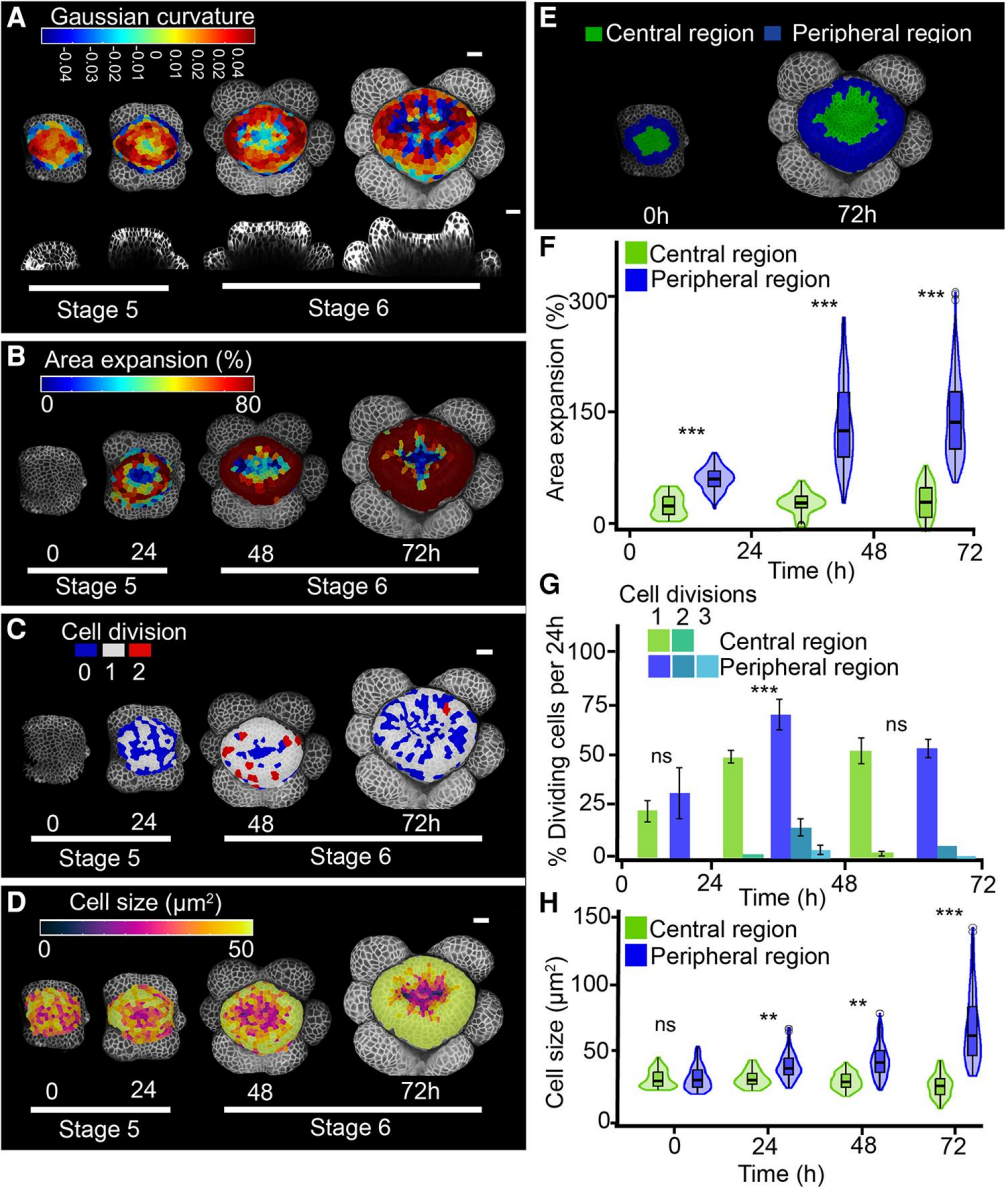
Cytokinin accelerates growth and division during gynoecium initiation in Arabidopsis. Floral meristems were treated with 10 μM of the synthetic cytokinin 6-benzylaminopurine and images were captured from early stage 5 to late stage 6 over 72 h at 24 h intervals. Floral stages are according to Smyth *et al*. (1990). (A–D) Heat maps of (A) Gaussian curvature, with the bottom row showing virtual longitudinal sections through the bud along its lateral axis, (B) area expansion between stages (C) cell divisions between stages, and (D) cell size. (E) Lineage tracking of the central and peripheral regions from early stage 5 to late stage 6. Scale bars are 20 µm. (F, G) Quantification of (F) area expansion, (G) cell divisions, and (H) cell size. Violin and box plots represent the 95% confidence intervals and the means (*n*=3 time-lapse series; error bars are SD). Bar plots show the percentage of cells that divided relative to the total number of cells in the central and peripheral regions per 24 h; this is further subdivided into cells that divided once, twice, or three times, as indicated in the key. Data from individual replicates are shown in Supplementary Fig. S3). Significant differences between regions were determined using Mann-Whitney *U*-tests: **P<0.001; ***P<0.0001; ns, not significant.

Both untreated and BAP-treated primordia displayed growth differences between the central region and the periphery (Fig. 1D; Fig. 2B, F; Supplementary Figs S2A, S3A, B G, H). However, BAP treatment further increased growth rates and curvature in the peripheral region and enhanced cell division in both regions as compared to the control (Fig. 1C, D; Fig. 2A, B). At early stage 5, epidermal cells in the FM were relatively small and homogeneous, with cell sizes ranging from 18–48 µm^2^ in the central region and 16–59 µm^2^ in the peripheral region (Fig. 2D, H; Supplementary Figs S2B, S3E, F K, L). From late stage 5, differences in cell size between the two regions became apparent: sizes in the central region remained within 10–64 µm^2^, while the peripheral region became significantly larger and exhibited a broader range of 15–229 µm^2^. Cell division also differed significantly between the two regions, with BAP treatment increasing the division rate in both the central and peripheral regions compared to untreated primordia (Figs 1E, I, Fig. 2C, G; Supplementary Fig. S3C, D I, J). This increase in cell division was reflected in cell numbers: in the central region, BAP-treated primordia increased from ∼21 cells at the start of treatment to 78 cells after 72 h, while in the peripheral region cell numbers rose from 44 to 216 cells (Supplementary Fig. S4A). In contrast, untreated primordia showed lower cell numbers, increasing from a mean of 11 cells to 19 in the central region and from 69 to 123 cells in the peripheral region after 72 h (Supplementary Fig. S4B).

These spatial differences in growth, cell size, and division rates were associated with enhanced peripheral bulging (positive curvature; Fig. 2A) and an increased diameter of the emerging gynoecium in BAP-treated samples. These growth alterations changed the geometry of the central region: the slow-growing region extended into both the lateral and medial regions, forming a cross-shaped pattern. As a result, the shape of the central invaginations was no longer medio–laterally elongated as in the untreated WT but became rounder or cross-shaped with a less-regular surface (Figs 1C, D, 2A, B; Supplementary Video S2).

Taken together, these results suggest that BAP treatment stimulated cell proliferation and expansion during carpel primordia establishment, resulting in increased primordia diameters, and altered patterning and architecture of the central invagination.

### Disruption of auxin transport decreases growth and division during gynoecium initiation

Polar auxin transport is critical for proper carpel initiation, gynoecium patterning, and development (e.g. Nemhauser *et al*., 2000; Ståldal, *et al*., 2008; Sorefan *et al*., 2009; Larsson *et al*., 2014; Moubayidin and Østergaard, 2014; Zúñiga-Mayo *et al*., 2014; Reyes-Olalde *et al*., 2017; Gómez-Felipe *et al*., 2024). Therefore, we used NPA, a well-characterized inhibitor of PIN-mediated auxin transport (Geldner *et al*., 2001; Noh *et al*., 2001), in order to examine its effects on cellular dynamics during gynoecium initiation.

**Fig. 3.**
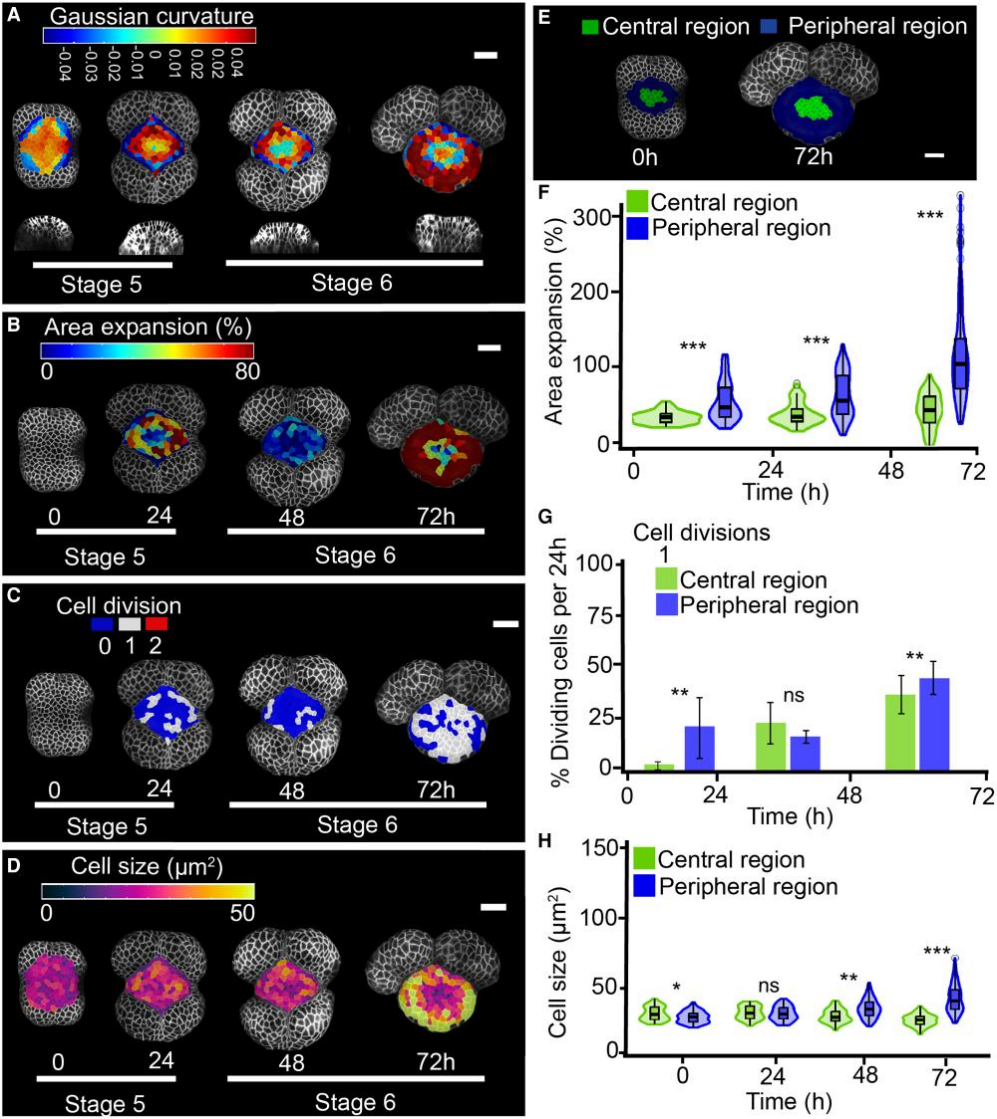
Disruption of auxin transport decreases growth and division during gynoecium initiation in Arabidopsis. Floral meristems were treated with 2 μM of N-1-naphthylphthalamic acid, an inhibitor of PIN-mediated auxin transport and images were captured from early stage 5 to late stage 6 over 72 h at 24 h intervals. Floral stages are according to Smyth *et al*. (1990). (A–D) Heat maps of (A) Gaussian curvature, with the bottom row showing virtual longitudinal sections through the bud along its lateral axis, (B) area expansion, (C) cell divisions, and (D) cell size. (E) Lineage tracing of central and peripheral regions from early stage 5 to late stage 6. Scale bars are 20 µm. (F–H) Quantification of (F) area expansion, (G) cell divisions, and (H) cell size; ‘1’ indicates that the cells underwent one division. Violin and box plots represent the 95% confidence intervals and the means (*n*=3 time-lapse series; error bars are SD). Bar-plots show the percentage of cells that divided relative to the total number of cells in the central and peripheral region per 24 h. Data from individual replicates are shown in Supplementary Fig. S5. Significant differences between regions were determined using Mann-Whitney *U*-tests: *P<0.01; **P<0.001; ***P<0.0001; ns, not significant.

As in the control, the epidermal cells in the FM were relatively small and homogeneous under NPA treatment, with sizes ranging from 15–39 µm^2^ in the central region and from 11–39 µm^2^ in the periphery at early stage 5 (Fig. 3D, H; Supplementary Figs S2B, S5E, F K, L). Cell sizes in the central region remained within a similar range throughout the live-imaging experiment: 13–36 µm^2^ at 24 h, 10–38 µm^2^ at 48 h, and 6–38 µm^2^ at 72 h. A noticeable difference between regions emerged at 72 h after treatment, when peripheral cells displayed a broader size range (7–101 µm^2^), in contrast to the consistently smaller cells in the central region. In line with these observations, higher cell division rates were detected in the peripheral region compared to the central region at both 24 and 72 h after treatment (Fig. 3C, G; Supplementary Fig. S5C, D I, J).

Although the overall pattern of growth and cell size in NPA-treated primordia resembled that of the untreated controls, with low activity in the central region and higher activity in the periphery (Figs 1D, Fig. 3B, F; Supplementary Fig. S5A, B G, H), the overall reduction in growth led to smaller primordia and a rounder morphology (Fig. 3B; Supplementary Video S3). Specifically, the slow-growing central region failed to maintain its lateral patterning and instead developed rounded invaginations, suggesting impaired tissue patterning at initiation (Fig. 3A, B). These results indicated that active auxin transport is required to establish proper central region architecture, and it affects the medio–lateral patterning.

### Complementary hormonal domains control carpel initiation

Auxin and cytokinin activities appear to occur in complementary domains during gynoecium initiation (Müller *et al*., 2017). To better understand the role of these hormones in regulating cellular growth, we first examined their spatial dynamics at the cellular level during carpel initiation. Specifically, we monitored the expression of the cytokinin-signaling reporter *TWO-COMPONENT OUTPUT SENSOR* (*TCSn*) (Zürcher *et al*., 2013) and the auxin-response reporter *DR5v2::nls-3xVenus* (Liao *et al*., 2015).

**Fig. 4.**
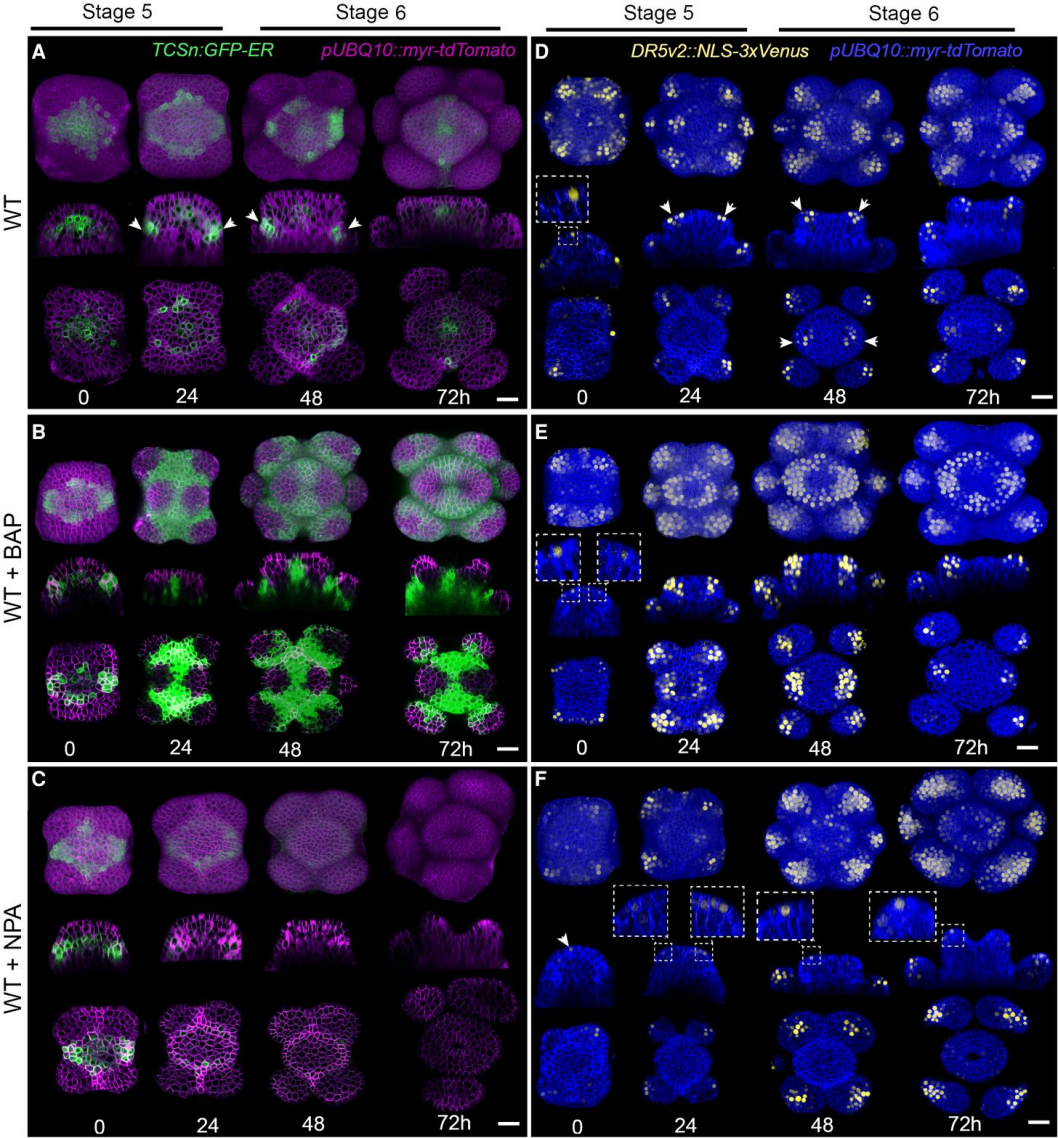
Complementary hormonal domains in the gynoecium control carpel initiation in Arabidopsis. (A–C) Expression patterns at 0–72 h of the *TCSn* cytokinin reporter during gynoecium initiation in (A) untreated (wild type, WT) samples, and in flowers treated with (B) 10 μM of the synthetic cytokinin 6-benzylaminopurine (BAP) or (C) 2 μM of N-1-naphthylphthalamic acid (NPA), an inhibitor of PIN-mediated auxin transport. The *TCSn* signal is in green and the TdTomato membrane marker is in purple. (D–F) Expression patterns of the *DR5v2* auxin reporter during gynoecium initiation in (D) the untreated samples, and in flowers treated with (E) BAP or (F) NPA. The *DR5v2* signal is in yellow and the membrane marker is in blue. The images show maximal signal projections of the floral bud (top rows), virtual longitudinal sections along the lateral axis of the buds (middle rows), and transverse sections ∼15–20 µm below the surface (bottom rows). The images are representative of *n*=5 replicates. Scale bars are 20 µm.

At the onset of stage 5, *TCSn* expression was detected broadly, particularly in the third and fourth cell layers at the center of the FM (Fig. 4A, 0 h). By the end of stage 5 (24 h), the signal decreased in intensity and became restricted to the meristem center, while increasing in the peripheral and basal domains to form a distinct halo-like pattern at the boundary between the emerging carpel and stamen primordia. At early stage 6, the cytokinin signaling region in the periphery split into four distinct basal domains, which started to disappear at late stage 6, while the *TCSn* signal persisted in the very center of the meristem (48–72 h).

At early stage 5, the *DR5v2* signal first appeared as two lateral foci in three or four epidermal cells, prior to any visible outgrowth, when the meristem still exhibited a dome-shaped structure (Fig. 4D, 0 h). Carpel primordia began to undergo morphological changes, developing rounded lateral edges, immediately following the emergence of these auxins signaling foci at late stage 5 (Fig. 4D, white arrows). At stage 6, *DR5v2* expression progressively expanded into the lateral inner layers (L1–L4), displaying a basipetal pattern (Fig. 4D, 48–72 h). Auxin internalization coincided with the outgrowth of the lateral edges of the carpel primordia at early stage 6 and became more pronounced by late stage 6. Auxin activity appeared to be spatially complementary to cytokinin activity throughout initiation, consistent with previous observations (Müller *et al*., 2017).

We next tested how cytokinin application altered this balance. Following exogenous treatment with the synthetic cytokinin BAP, *TCSn* expression expanded across the medial domain, including the epidermis (Fig. 4B). The signal intensity was markedly stronger than in the control, although expression remained absent from the lateral carpel primordia. *DR5v2* expression also strongly increased and broadened under BAP treatment. It formed a ring-like epidermal domain but remained consistently excluded from the central region (Fig. 4E). Auxin activity also expanded into inner tissues, both laterally and in depth, at the sites of emerging carpel primordia. Thus, BAP treatment enhanced both cytokinin signaling and auxin activity, but the expanded domains remained spatially complementary.

By contrast, NPA treatment strongly reduced the *TCSn* signal beginning at late stage 5, and the it became nearly undetectable by late stage 6 (Fig. 4C). *DR5v2* expression was also reduced and it failed to internalize into lateral tissues as seen in the untreated controls (Fig. 4F). Instead, it spread broadly across the epidermis of the carpel primordia, losing its normal localized distribution.

Together, these results indicated that the cytokinin and auxin responses were spatially complementary during carpel primordia initiation: cytokinin activity was enriched in the central and basal domains, while auxin accumulated laterally to guide growth differences and tissue patterning. Perturbing this balance via exogenous BAP or NPA treatments altered the geometry, size, and shape of the emerging carpel primordia.

## Discussion

**Fig. 5.**
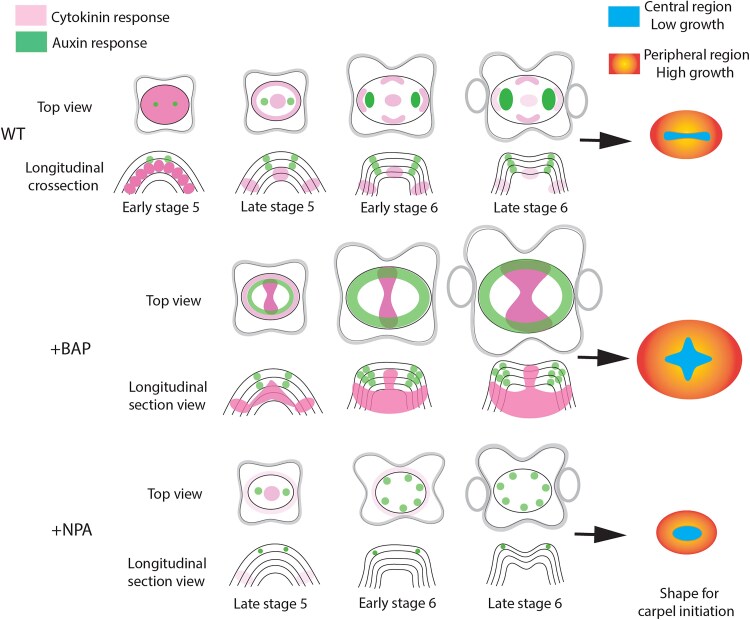
Coordinated cytokinin and auxin responses are required for proper carpel primordia growth and shape establishment in Arabidopsis. Schematic representations of carpel primordia initiation are shown for the untreated wild type (WT), and for samples treated with the synthetic cytokinin 6-benzylaminopurine (+BAP), or with N-1-naphthylphthalamic acid (+NPA), an inhibitor of PIN-mediated auxin transport. In the wild type, both hormones show defined and complementary distribution patterns, leading to the generation of faster-growing peripheral regions (yellow) and a slow-growing elongated central region (blue). Upon BAP treatment, the cytokinin response expands in the inner layers, and auxin distribution broadens from the epidermis into inner tissues, resulting in a larger carpel primordium and an expanded central region. In contrast, NPA treatment reduces the cytokinin signal in the inner layers and prevents auxin transport to the inner layers, leading to a smaller and less-defined carpel primordium. The diagrams at the right summarize the final shapes observed under each condition. Floral stages are according to Smyth *et al*. (1990).

In this study, we conducted a quantitative analysis of carpel primordium establishment in Arabidopsis, based on growth measurements obtained from live confocal imaging (Fig. 1). Unlike earlier qualitative descriptions (Smyth *et al*., 1990; Bossinger and Smyth, 1996; Müller *et al*., 2017), our data provide a dynamic, spatially resolved view of gynoecium initiation. By integrating quantification of cell-level growth with hormone reporter analyses and pharmacological treatments, we demonstrate that auxin and cytokinin display distinct activity domains in the epidermis and inner layers. These hormones have opposing effects on growth and cell division, which together shape the geometry of the emerging gynoecium (Fig. 5).

Our quantification revealed that ∼60 epidermal cells contributed to the gynoecium primordium at early stage 5, with each carpel primordium containing ∼30 epidermal cells. This is strikingly similar to stamen primordia, which have been reported to comprise 28 epidermal cells at inception (Silveira *et al*., 2022). By comparison, earlier histological studies estimated ∼337 total cells per carpel primordium at stage 6 (Crone and Lord, 1994; Hill and Lord, 1989; Jenik and Irish, 2000), probably reflecting the cumulative contribution of all cell layers as well as a more advanced developmental stage.

Furthermore, our results revealed that differences in cell growth rate, division, and size between peripheral and central regions underlie the formation of the tube-like gynoecium primordium during early development. In the central region, small, slowly growing cells were aligned along the medio–lateral axis, suggesting that this population serves as a structural scaffold supporting the expansion of the faster-growing peripheral cells, particularly in the lateral regions destined to form the valves (Fig. 1D). Comparable growth dynamics have been reported in *Aquilegia coerulea*, where rapid peripheral expansion accompanies carpel primordium initiation (Min *et al*., 2022), suggesting that this mechanism might be conserved across species. This observation also supports the view that carpels, like other aerial organs, are modified leaf structures (Pelaz *et al*., 2001). In developing leaf primordia, growth is typically asymmetric, with the abaxial side expanding more rapidly than the adaxial side (Kierzkowski *et al*., 2012). A similar asymmetry, characterized by faster growth on the abaxial side, is likewise conserved during early carpel development.

As expected, the central domain contributed to the future carpel margin meristem (CMM), which becomes morphologically visible at stage 7 as two adaxial medial ridges (Nole-Wilson *et al*., 2010; Wynn *et al*., 2011; Reyes-Olalde and de Folter, 2019). Although a specific marker for meristematic cells in carpel development is still lacking, the central cells aligned along the medio–lateral axis exhibited meristematic features, including small size and low division rates. This cell population was already present at stage 6 (Fig. 1C–F), suggesting that a pre-CMM is established prior to visible morphological differentiation. The presence of a pre-CMM at stage 6 suggests the formation of a meristematic domain at earlier stages. In carpel primordia, cytokinin signaling has previously been detected at stage 5 using the *TCSn* reporter, with signals observed in apical and subapical medial cells located ∼10–15 µm below the apex (Müller *et al*., 2017). Cytokinin is a key regulator of meristem function, promoting cell proliferation and sustaining the undifferentiated state in the shoot apical meristem and FM (e.g. Shani *et al*., 2006; Gordon *et al*., 2009; Bartrina *et al*., 2011; Scofield *et al*., 2013; Schaller *et al*., 2014). Our analyses were consistent with these earlier findings and provide higher spatial resolution. At early stage 5, we observed *TCSn* expression in the third and fourth cell layers of the medial domain, extending into the basal-lateral domain and even reaching the epidermis (Fig. 4A, 0 h). From late stage 5 onward, *TCSn* expression became restricted to a small population of medial cells. Application of BAP induced an expansion of the *TCSn* signal in the inner layers, which also extended to the epidermis (Fig. 4B). These results suggest that cytokinin plays a crucial role in establishing the putative meristematic domain of the carpel primordium and in the formation of the pre-CMM. Notably, this *TCSn* expression pattern resembles that observed in the third and fourth layers of the shoot apical meristem (Murray *et al*., 2012; Fig. 1D).

Overall, the enhanced *TCSn* response under BAP treatment coincided with increased proliferation in the central region, maintenance of small cell size, and reduced overall growth, features that collectively disrupted the normal medio–lateral growth pattern. In contrast, the enhanced *DR5v2* response in the lateral domain was associated with increased peripheral growth and proliferation. These complementary spatial distributions suggest that cytokinin and auxin regulate distinct proliferative zones within the primordium: cytokinin signaling might sustain a meristematic state in the central domain, whereas auxin promotes growth and expansion in the periphery.

Auxin functions as a morphogen, establishing spatial patterns critical for organ positioning and development (e.g. Nemhauser *et al*., 2000; Friml *et al*., 2002, 2003; Marchant *et al*., 2002; Benková *et al*., 2003; Reinhardt *et al*., 2003; Heisler *et al*., 2005; Scarpella *et al*., 2006). Consistent with this, inhibition of auxin transport with NPA at early stages produced smaller, rounder carpel primordia with enhanced peripheral growth and loss of the slow-growing medial cells that normally form elongated invagination (Fig. 4).

Under NPA treatment, we observed that the two characteristic medial auxin maxima were lost (Fig. 4A, B, E, F). Instead, the *DR5v2* auxin-responsive reporter showed a patchy, non-homogeneous distribution throughout the epidermal layer (Fig. 4E, F). These observations suggest that the increased peripheral growth resulted from auxin accumulating in the outer epidermis. Furthermore, the absence of laterally extended slow-growing cells in the center, combined with the rounder shape of the primordium, could underlie the ‘valveless’ gynoecium phenotype observed at later stages (Nemhauser *et al*., 2000; Sohlberg *et al*., 2006; Ståldal *et al*., 2008; Zúñiga-Mayo *et al*., 2014; Gómez-Felipe *et al*., 2024). This alteration of the normal medio–lateral patterning correlated with an almost undetectable *TCSn* signal in the central region under NPA treatment (Fig. 4E, F). Taken together, these results indicated that disrupting polar auxin transport early on affects the initial patterning of the pre-CMM.

Notably, previous studies have shown that perturbing auxin transport at early stages interferes with lateral procambium formation, thereby affecting both the medio–lateral and apical–basal patterning of the gynoecium. This ultimately compromises the development of internal tissues (placenta, ovules, transmitting tract) that arise from the CMM, leading to defects in these structures (Nemhauser *et al*., 2000; Sohlberg *et al*., 2006; Ståldal *et al*., 2008; Larsson *et al*., 2014; Zúñiga-Mayo *et al*., 2014; Gómez-Felipe *et al*., 2024). Moreover, a high local auxin concentration is known to be required for proper cell proliferation and morphogenesis of the style (Sohlberg *et al*., 2006; Ståldal *et al*., 2008).

Our findings also support and expand on the model proposed by Sessions *et al*. (1997) and later modified by Nemhauser *et al*. (2000), in which carpel primordia are patterned through early specification of boundaries within the dome of cells. Taken together, our results support a model in which carpel primordia establishment proceeds through two sequential phases. First, growth differences between central and peripheral regions are guided by complementary cytokinin and auxin domains: cytokinin maintains a meristematic state in the central domain, while auxin promotes lateral expansion in the periphery. Second, apical–basal patterning is elaborated and stabilized through the formation of a basipetal auxin gradient and cytokinin-dependent development of the internal CMM. This framework integrates and extends the classic models of Sessions *et al*. (1997) and Nemhauser *et al*. (2000) by linking spatial growth dynamics with hormone signaling at cellular resolution (Fig. 5). By uncovering how auxin and cytokinin coordinate growth behavior during early gynoecium initiation, our study provides a mechanistic basis for understanding carpel morphogenesis and opens new avenues for exploration, particularly how internal tissue layers are reorganized in 3D to shape gynoecium development.

## Supplementary Material

eraf535_Supplementary_Data

## Data Availability

The raw data used for quantification are openly available to download from the Open Science Framework repository at https://osf.io/azpt8/.

## Supplementary data

The following supplementary data are available at *JXB* online.

Fig. S1. Additional biological replicates for carpel primordia initiation in the control samples shown in Fig. 1.

Fig. S2. Cell growth and size dynamics of carpel primordia in control plants and under BAP or NPA treatment.

Fig. S3. Additional biological replicates for carpel primordia initiation in the BAP-treated samples shown in Fig. 2.

Fig. S4. Cell numbers the central and peripheral regions in control, BAP-treated, and NPA-treated primordia.

Fig. S5. Additional biological replicates for carpel primordia initiation in the NPA-treated samples shown in Fig. 3.

Video S1. Capel primordium of the wild type control at stage 6.

Video S2. Carpel primordium at stage 6 treated with BAP at 72 h after treatment.

Video S3. Carpel primordia at stage 6 treated with NPA at 72 h after treatment.

## Abbreviations

BAP: N6-benzylaminopurine
CMM: carpel margin meristem
FM: floral meristem
NPA: N-1-naphthylphthalamic acid
