# Supplementary material for: Auxin and cytokinin regulate growth dynamics underlying carpel initiation in Arabidopsis
Source: J Exp Bot. 2025 Dec 12;77(6):1743–55. doi: 10.1093/jxb/eraf535 (PMC13016931; doi:10.1093/jxb/eraf535)
Supplement: eraf535_Supplementary_Data [file eraf535_supplementary_data.zip › jexbot316493-file001.pdf]

Auxin and cytokinin regulate growth dynamics underlying carpel initiation

Andrea Gómez-Felipe[1,3], Stefan de Folter[2*], Daniel Kierzkowski[1*]

[1] Institut de Recherche en Biologie Végétale, Département de Sciences Biologiques, Université de Montréal, 4101 Sherbrooke St E, Montréal, QC, H1X 2B2 Canada

[2] Unidad de Genómica Avanzada (UGA-Langebio), Centro de Investigación y de Estudios Avanzados del Instituto Politécnico Nacional (Cinvestav), CP 36824 Irapuato, México

[3] Present address: Department of Biology, Indiana University, Bloomington, IN 47405

*Correspondence: stefan.defolter@cinvestav.mx and daniel.kierzkowski@umontreal.ca

SUPPLEMENTARY FIGURES

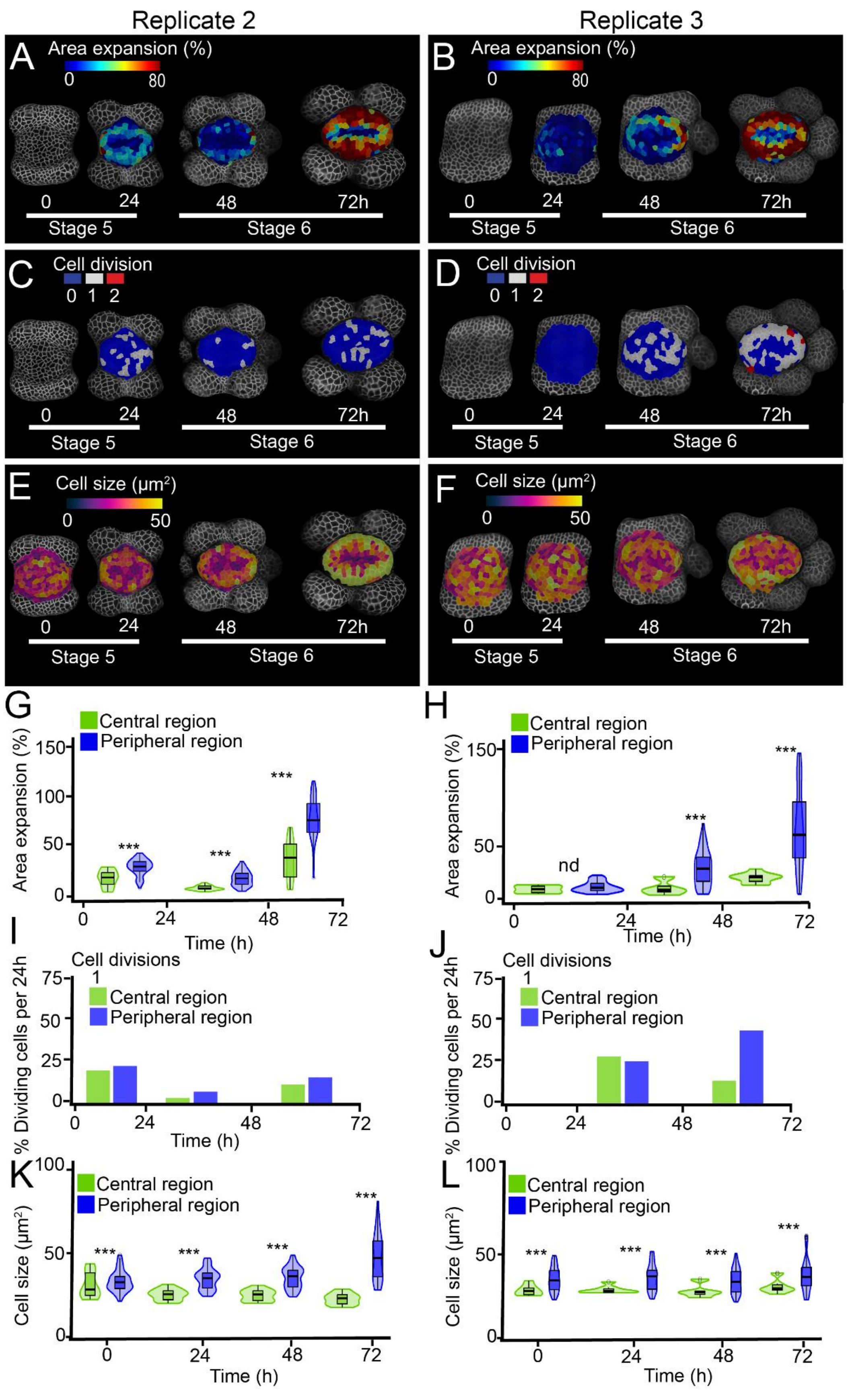

Replicate 2
Replicate 3
A
Area expansion (%)
0
80
0
24
48
72h
Stage 5
Stage 6
B
Area expansion (%)
0
80
0
24
48
72h
Stage 5
Stage 6
C
Cell division
0 1 2
0
24
48
72h
Stage 5
Stage 6
D
Cell division
0 1 2
0
24
48
72h
Stage 5
Stage 6
E
Cell size (µm²)
0
50
0
24
48
72h
Stage 5
Stage 6
F
Cell size (µm²)
0
50
0
24
48
72h
Stage 5
Stage 6
G
Central region
Peripheral region
Area expansion (%)
Time (h)
***
H
Central region
Peripheral region
Area expansion (%)
nd
***
Time (h)
I
Cell divisions
1
Central region
Peripheral region
% Dividing cells per 24h
Time (h)
J
Cell divisions
1
Central region
Peripheral region
% Dividing cells per 24h
K
Central region
Peripheral region
Cell size (µm²)
***
Time (h)
L
Central region
Peripheral region
Cell size (µm²)
***
Time (h)

21

Figure S1. Biological replicates for wild type carpel primordia initiation. (A-F) Heat maps area expansion **(A-B)**; cell divisions **(C-D)**; and cell size **(E-F)**. **(G-L)** Quantification of area expansion **(G-H)**; cell divisions **(I-J)**; and cell sizes **(K-L)**. Violin and box plots represent 95% of the data distribution; solid lines indicate the mean. Bar plots show the percentage of cells that divided relative to the total number of cells in the central and peripheral region per 24h; this number was further subdivided into cells that divided once, twice, or three times, as represented by the color of the bar. Data shown in this figure correspond to each independent time-lapse series. Statistical significance with Mann-Whitney U test. *, **, ***, p<0.01, p<0.001 and p<0.0001 respectively. Scale bars = 20 μm.

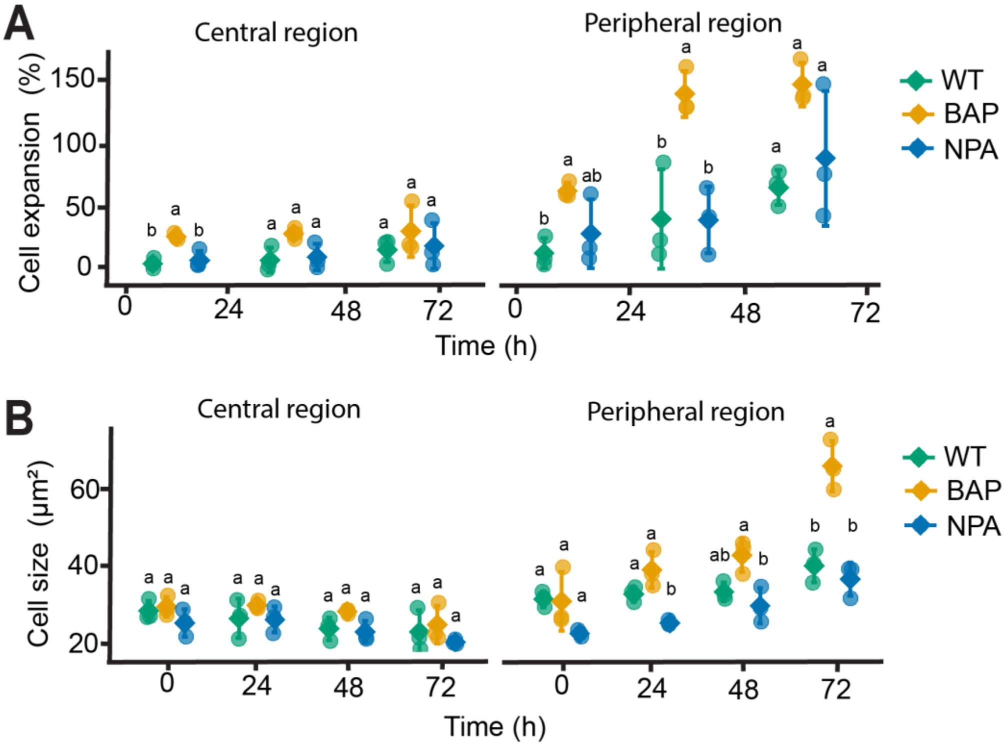

Figure S2. Comparison of growth and size dynamics of carpel primordia in WT and under BAP or NPA treatment. (A-B) Quantification of cell expansion **(A)** and cell sizes **(B)** in the central and peripheral regions under three conditions: WT (control), BAP, and NPA. Each point represents the mean value per biological replicate (n=3 per treatment). Error bars indicate standard deviation. Statistical significance was assessed using one-way ANOVA followed by Tukey's HSD post-hoc test. Groups sharing the same letter are not significantly different ($p>0.05$).

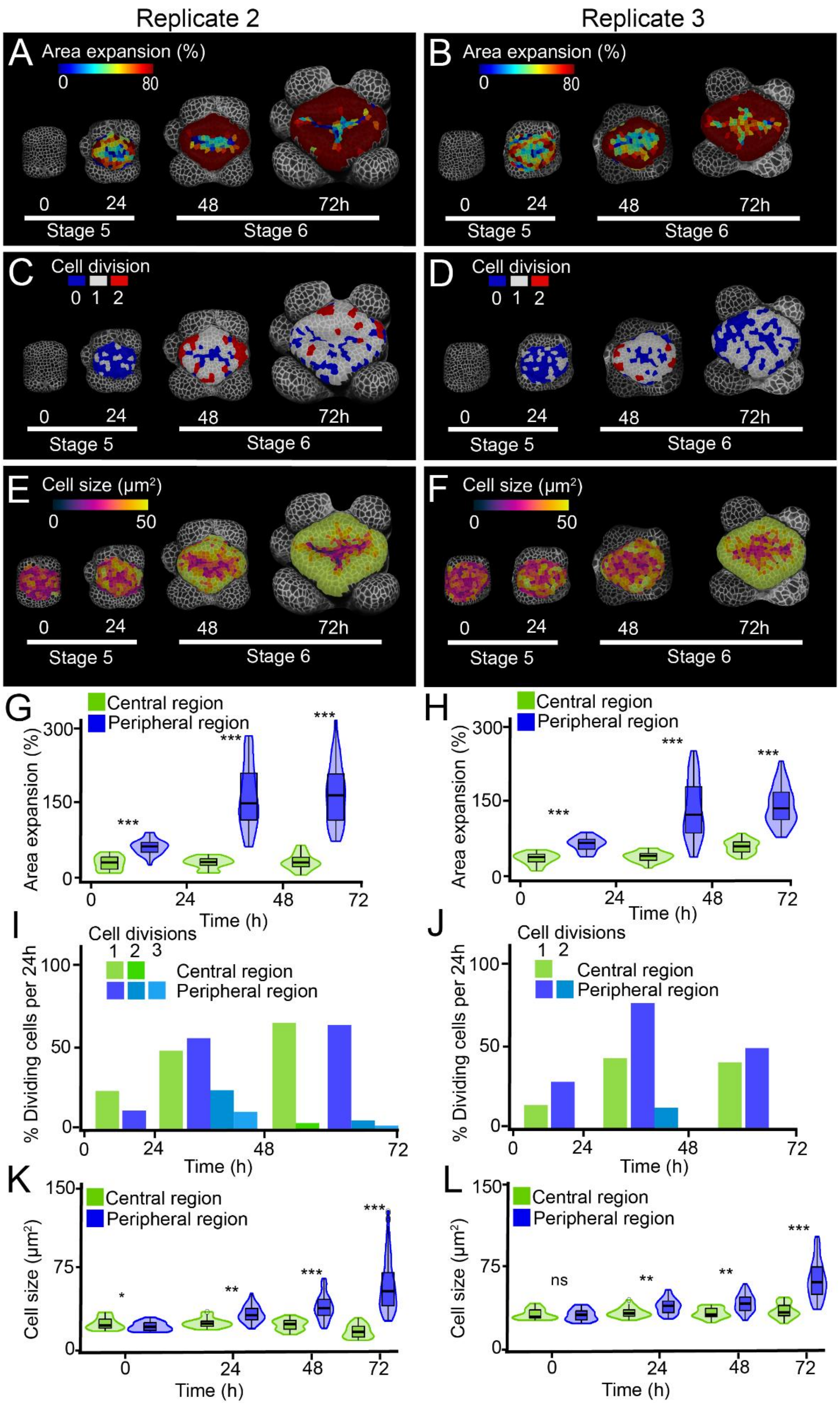

Replicate 2
Replicate 3
A Area expansion (%)
0 80
0 24 48 72h
Stage 5 Stage 6
B Area expansion (%)
C Cell division
0 1 2
D Cell division
E Cell size (μm²)
0 50
F Cell size (μm²)
G Central region
Peripheral region
Area expansion (%)
Time (h)
H
I Cell divisions
1 2 3
% Dividing cells per 24h
J
K Cell size (μm²)
L
ns

42

Figure S3. Biological replicates for carpel primordia treated with BAP. (A-F) Heat maps area expansion **(A-B)**; cell divisions **(C-D)**; and cell size **(E-F)**. **(G-L)** Quantification of area expansion **(G-H)**; cell divisions **(I-J)**; and cell sizes **(K-L)**. Violin and box plots represent 95% of the data distribution; solid lines indicate the mean. Bar plots show the percentage of cells that divided relative to the total number of cells in the central and peripheral region per 24h; this number was further subdivided into cells that divided once, twice, or three times, as represented by the color of the bar. Data shown in this figure correspond to each independent time-lapse series. Statistical significance with Mann-Whitney U test. *, **, ***, $p<0.01$, $p<0.001$ and $p<0.0001$ respectively. Scale bars = 20 μm.

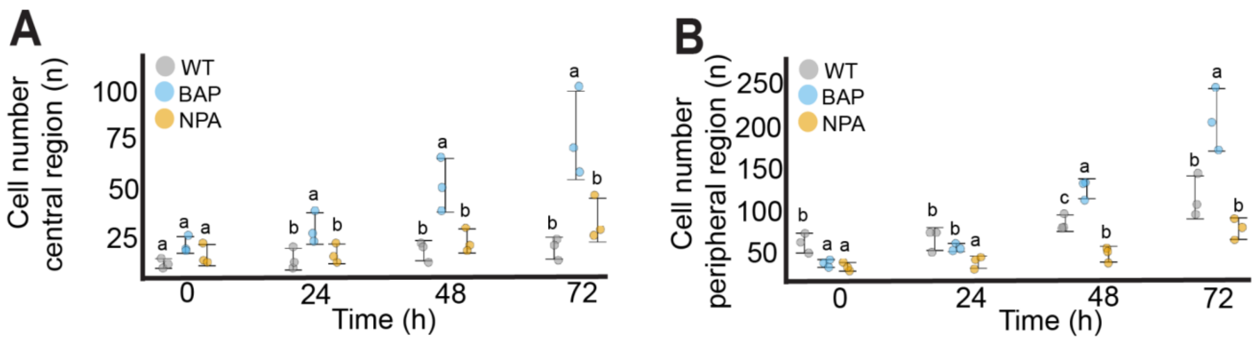

Figure S4. Cell number in wild type, BAP-treated, and NPA-treated primordia. (A-B) Total cell number in the central **(A)** and peripheral **(B)** regions. Each point represents the mean value of an individual biological replicate (n=3 per treatment). Error bars indicate standard deviation. Statistical significance was evaluated using one-way ANOVA followed by Tukey's HSD post-hoc test. Groups sharing the same letter are not significantly different ($p>0.05$).

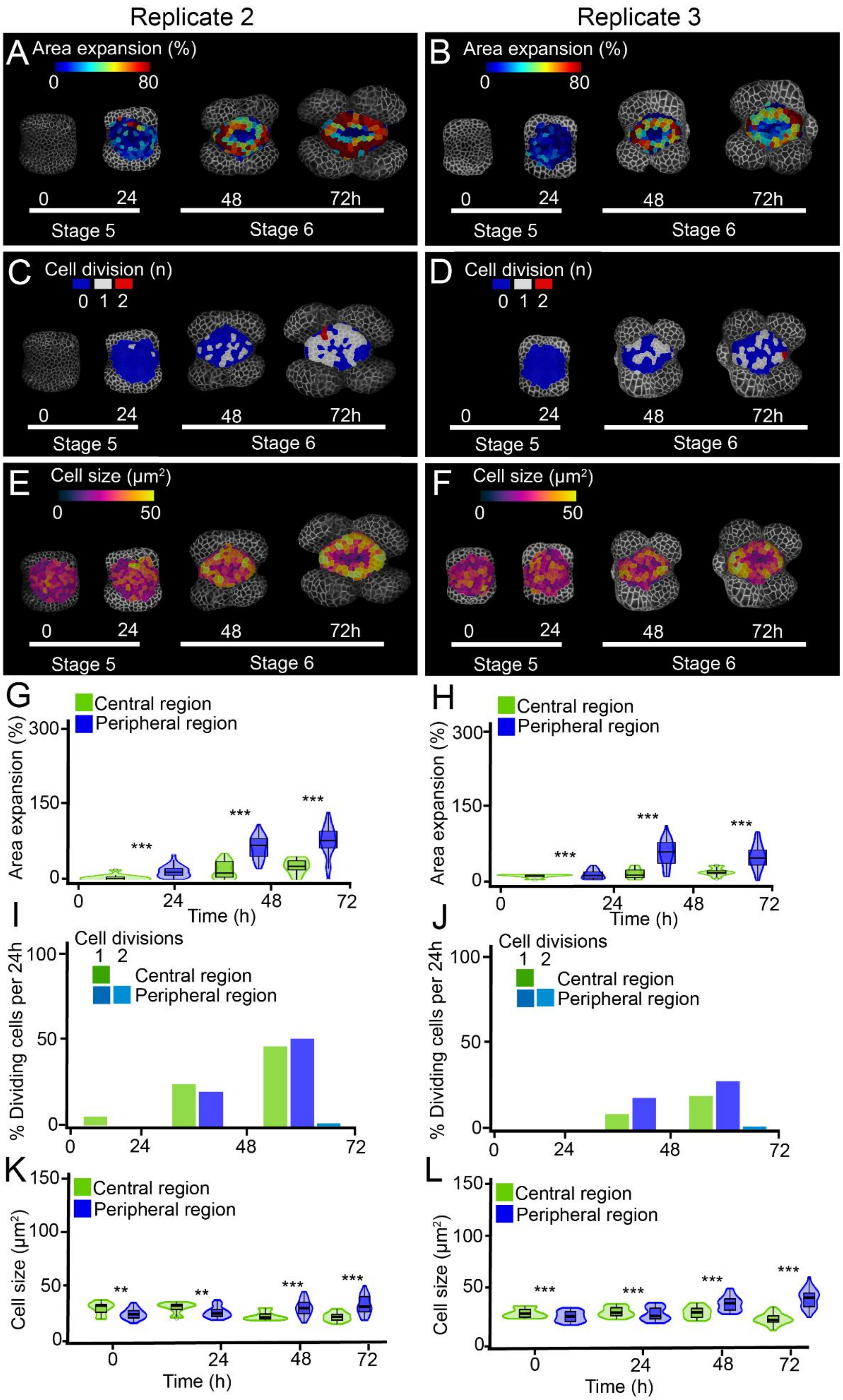

Replicate 2
Replicate 3
A Area expansion (%)
0 80
0 24 48 72h
Stage 5
Stage 6
B Area expansion (%)
0 80
0 24 48 72h
Stage 5
Stage 6
C Cell division (n)
0 1 2
0 24 48 72h
Stage 5
Stage 6
D Cell division (n)
0 1 2
0 24 48 72h
Stage 5
Stage 6
E Cell size (μm²)
0 50
0 24 48 72h
Stage 5
Stage 6
F Cell size (μm²)
0 50
0 24 48 72h
Stage 5
Stage 6
G
Central region
Peripheral region
Area expansion (%)
Time (h)
H
Central region
Peripheral region
Area expansion (%)
Time (h)
I
Cell divisions
1 2
Central region
Peripheral region
% Dividing cells per 24h
J
Cell divisions
1 2
Central region
Peripheral region
% Dividing cells per 24h
K
Central region
Peripheral region
Cell size (μm²)
Time (h)
L
Central region
Peripheral region
Cell size (μm²)
Time (h)

67

Figure S5. Biological replicates for carpel primordia treated with NPA. (A-F) Heat maps area expansion **(A-B)**; cell divisions **(C-D)**; and cell size **(E-F)**. **(G-L)** Quantification of area expansion **(G-H)**; cell divisions **(I-J)**; and cell sizes **(K-L)**. Violin and box plots represent 95% of the data distribution; solid lines indicate the mean. Bar plots show the percentage of cells that divided relative to the total number of cells in the central and peripheral region per 24h; this number was further subdivided into cells that divided once, twice, or three times, as represented by the color of the bar. Data shown in this figure corresponds to each independent time-lapse series. Statistical significance with Mann-Whitney U test. *, **, ***, $p<0.01$, $p<0.001$ and $p<0.0001$ respectively. Scale bars = 20 μm.
